# *Nigella sativa* supplementation improves cardiometabolic indicators in population with prediabetes and type 2 diabetes mellitus: A systematic review and meta-analysis of randomized controlled trials

**DOI:** 10.3389/fnut.2022.977756

**Published:** 2022-08-11

**Authors:** Saeede Saadati, Kaveh Naseri, Omid Asbaghi, Khadijeh Abhari, Pangzhen Zhang, Hua-Bin Li, Ren-You Gan

**Affiliations:** ^1^Department of Medicine, School of Clinical Sciences, Monash University, Melbourne, VIC, Australia; ^2^Gastroenterology and Liver Diseases Research Center, Research Institute for Gastroenterology and Liver Diseases, Shahid Beheshti University of Medical Sciences, Tehran, Iran; ^3^Cancer Research Center, Shahid Beheshti University of Medical Sciences, Tehran, Iran; ^4^Department of Food Science and Technology, National Nutrition and Food Technology Research Institute, Faculty of Nutrition Sciences and Food Technology, Shahid Beheshti University of Medical Sciences, Tehran, Iran; ^5^School of Agriculture and Food, Faculty of Veterinary and Agricultural Sciences, University of Melbourne, Parkville, VIC, Australia; ^6^Guangdong Provincial Key Laboratory of Food, Nutrition and Health, Department of Nutrition, School of Public Health, Sun Yat-sen University, Guangzhou, China; ^7^Research Center for Plants and Human Health, Institute of Urban Agriculture, National Agricultural Science and Technology Center, Chinese Academy of Agricultural Sciences, Chengdu, China

**Keywords:** diabetes mellitus, prediabetes, meta-analysis, *Nigella sativa*, cardiometabolic, lipid profile, glycemic homeostasis

## Abstract

**Objective:**

*Nigella sativa (N. sativa)* from the family *Ranunculaceae* has medicinal properties. Previous studies have reported promising findings showing that *N. sativa* may benefit cardiometabolic health; however, current evidence on its cardiometabolic effects on those with prediabetes and type 2 diabetes mellitus (T2DM) is still unclear. Hence, we conducted a systematic review and meta-analysis to assess the efficacy of *N. sativa* on cardiometabolic parameters in population with prediabetes and T2DM.

**Methods:**

PubMed/Medline, ISI Web of Science, Scopus, and Cochrane library were systematically searched up to June 20, 2022. Meta-analyses using random-effects models were used.

**Results:**

Eleven randomized controlled trials (RCTs) were included in the meta-analysis. *N. sativa* intervention resulted in significant changes in fasting plasma glucose (FPG), hemoglobin A1c (HbA1c), total cholesterol (TC), low-density lipoprotein cholesterol (LDL-C), c-reactive protein (CRP), and malondialdehyde (MDA), without overall changes in glucose levels after oral glucose tolerance test (OGTT), fasting insulin, homeostatic model assessment of insulin resistance (HOMA-IR), triglyceride, high-density lipoprotein cholesterol (HDL-C), and body mass index (BMI) when compared with the control group. In subgroup analyses, *N. sativa* supplementation enhanced serum levels of HDL-C in subjects with baseline HDL-C lower than 40 mg/dL. Furthermore, HOMA-IR and BMI values decreased in the *N. sativa*-supplemented group compared with the control group, when the length of follow-up was more than 8 weeks and the dose was more than 1 g/day for *N. sativa* supplementation, respectively.

**Conclusion:**

Our findings indicate that *N. sativa* supplementation may effectively improve cardiometabolic profiles in individuals with prediabetes and T2DM.

## Introduction

In accordance with the reports from the latest Global Burden of Disease (GBD 2016), 72.3% of the mortality rate is attributed to non-communicable diseases (NCDs) (1). Type 2 diabetes mellitus (T2DM) is among the most prevalent NCDs with the global estimates up to 463 million adults in 2019 which is predicted to increase to 642 million people by 2040 (2, 3). Moreover, there are 318 million individuals with prediabetes as a marker for T2DM development (4, 5). It is particularly noteworthy that its prolonged nature, accompanied by diabetes-associated complications, leading T2DM to one of the most costly disease categories (6). Insulin resistance and cardiovascular risk factors, including obesity, hyperlipidemia, and hypertension, are the hallmarks of T2DM pathogenesis (7–9). Given the epidemic of diabetes-related cardiovascular events and economic burden of T2DM, exploring cost-effective and promising therapeutic approaches should be of substantial importance (6–10).

Today, several medications are marketed to deal with the complications of diabetes. However, some standard drugs, such as biguanides, meglitinides, thiazolidinedione, etc. cause side effects such as nausea, bloating, stomach pain, dark urine, and liver problems (11). Therefore, complementary and alternative medicine (CAM) has provided the opportunity to manage diabetes along with lifestyle interventions and nutrition therapies and also make patients with T2DM to investigate a surrogate therapy to the chemical medications (12, 13). *Nigella sativa (N. sativa)* from the family *Ranunculaceae* (buttercup), commonly called black caraway, black cumin, nigella, or kalonji, is a medicinal food with a wide range of health benefits, such as a gastro-protective, hepato-protective, anti-diabetic, antihypertensive, bronchodilator, immunomodulatory, anti-inflammatory, anti-hyperlipidemic, antioxidant, and anticancer effects (14–17).

Numerous studies proposed *N. sativa* as an adjuvant therapy in diabetes control, since it revealed a significant reduction in glucose values following oral glucose tolerance test (OGTT), fasting plasma glucose (FPG) and hemoglobin A1c (HbA1c) levels, and insulin resistance, and also an escalation in serum fasting insulin concentrations (18–20), while recent research reported that *N. sativa* supplementation increased the levels of glycemic control components (21). Furthermore, it has been demonstrated that *N. Sativa* extract could increase the high-density lipoprotein cholesterol (HDL-C) concentration through rising the activity of plasma lecithin cholesterol acetyltransferase (LCAT), and it could also exert its antioxidant effects by enhancing the activities of antioxidant enzymes, including catalase and glutathione peroxidase, leading to its potential efficacy in amelioration of atherosclerosis (22, 23). However, there are still strong pieces of evidence suggesting null effects of *N. sativa* on biomarkers of inflammation (21, 24).

Considering the discrepancies across all the available evidence, the efficacy of *N. sativa* on cardiometabolic parameters in subjects with prediabetes and T2DM is still unclear. In order to fill this knowledge gap, we conducted an in-depth systematic review and meta-analysis based on high-quality randomized controlled trials (RCTs) to evaluate the effects of *N. sativa* consumption on cardiovascular risk factors in individuals with prediabetes and T2DM.

## Methods

We conducted and reported the current systematic review and meta-analysis according to the Preferred Reporting Items for Systematic Review and Meta-analysis (PRISMA) checklist (25).

### Data sources and search strategy

Electronic databases (PubMed/MEDLINE, Scopus, Web of Science, and Cochrane library) were used to identify studies until June 20, 2022. A search strategy was implemented using the following keywords: (“Black cumin” OR “*Nigella sativa*” OR “black seed” OR “black caraway” OR “Roman coriander” OR “kalonji” OR “fennel flower” OR “pungent seeds”) AND (Intervention OR “controlled trial” OR randomized OR random OR randomly OR placebo OR “clinical trial” OR “randomized clinical trial” OR RCT OR trials OR “Cross-Over Studies” OR “Cross-Over” OR “Cross-Over Study” OR parallel OR “parallel study” OR “parallel trial”) AND (“diabetes” OR “type 2 diabetes mellitus” OR “T2DM” OR “type 2 diabetes” OR “T2D” OR “prediabetes”). Supplementary Table 1 lists search terms used across various databases. No date restrictions were applied, however, only English-language articles were eligible for inclusion. We screened the bibliographies of relevant studies and systematic reviews identified through the search strategy for additional studies. Data were requested from corresponding authors if the required data for meta-analysis were not reported.

### Study selection and eligibility criteria

For further screening, endnote software was used to save records from electronic and manual searches. Researchers independently evaluated the titles and abstracts of all articles identified in the initial search (S.S. and K.N.). Discussion with a third reviewer (O.A.) resolved the disagreement regarding full-text eligibility. The eligibility of articles was determined by the Population, Intervention, Comparison, Outcomes, and Study Design (PICOS) framework (Table 1). Eligibility for the studies included: (1) Population: adult subjects (≥18 years old) and with physician's diagnosis of impaired glucose tolerance or prediabetes or T2DM; (2) Intervention: administration of *Nigella sativa* in different chemical forms including oil, capsule, and tablet; (3) Comparators: comparison with placebo, any pharmacological or non-pharmacological intervention(s), or usual care; (4) Outcomes: those which reported mean changes and their standard deviations (SDs) of BMI, glycemic control parameters (FPG, OGTT, HbA1c, fasting insulin, and HOMA-IR), lipid profile components (TG, TC, LDL-C, and HDL-C), c-reactive protein (CRP), and malondialdehyde (MDA) during the trial for both intervention and control groups or provided the information required to calculate those effect sizes; and (5) Study design: being an RCT in either parallel or cross-over design. Studies were excluded from this investigation if they: (1) included *Nigella sativa* as a part of a complex intervention; (2) lacked suitable control; (3) had no viable endpoint data in *Nigella sativa* or control groups; (4) were carried out on pregnant women, children, or animals, and (5) were performed <4 weeks in duration. In addition, gray literature, conference abstracts, protocols, and unpublished studies were excluded.

**Table 1:** Population, intervention, comparison, outcomes, and study design (PICOS) criteria for inclusion of studies.

**Parameter**	**Inclusion criteria**
Population	Individuals older than 18 years and with physician's diagnosis of impaired glucose tolerance or prediabetes or T2DM
Intervention	Administration of *Nigella sativa* in different chemical forms including oil, capsule, tablet, and powder
Comparator	Comparison with placebo, usual care, or any pharmacological or non-pharmacological intervention(s)
Outcome	Those which reported mean changes and their standard deviations (SDs) of BMI, glycemic control parameters (FPG, insulin, HbA1c, OGTT, and HOMA-IR), lipid profile components (TG, TC, LDL-C, and HDL-C), CRP, and MDA throughout the trial for both intervention and control groups or presented required information for calculation of those effect sizes
Study design	Being an RCT in either parallel or cross-over design

T2DM, type 2 diabetes mellitus; SDs, standard deviations; BMI, body mass index; FPG, fasting plasma glucose; HbA1c, hemoglobin A1c; OGTT, oral glucose tolerance test; HOMA-IR, homeostatic model assessment of insulin resistance; TG, triglyceride; TC, total cholesterol; LDL-C, low-density lipoprotein cholesterol; HDL-C, high-density lipoprotein cholesterol; CRP, C-reactive protein; MDA, Malondialdehyde; RCT, randomized controlled trial.

### Data extraction

Two independent investigators extracted the following information from each eligible clinical trial: study author; publication year; study location; study design; the number of participants; participants' ethnicity, age, comorbidities, body mass index; the type, dose, duration, and frequency of the intervention; and the study results [mean or median with SDs, standard errors (SEs), 95% CIs, or interquartile ranges (IQRs)] at study baseline, post-intervention, and/or changes between baseline and post-intervention. We converted data from each endpoint when it was reported in different units.

### Quality assessment

In order to determine whether the included RCTs were likely to be biased, we used the Cochrane Risk of Bias Tool (26). The quality of each publication was evaluated by two independent authors using the following seven domains: (1) random sequence generation. (2) allocation concealment, (3) selective outcome reporting, (4) blinding of participants and personnel, (5) detection bias (blinding of data analyzers), (6) incomplete outcome data, and (7) other probable sources of biases. Each article was assigned either low-risk (L) or high-risk (H) bias label according to the Cochrane Handbook recommendations (Supplementary Table 2).

The Grading of Recommendations, Assessment, Development, and Evaluation (GRADE) method was used to assess the quality of evidence for each outcome (27). Each outcome was graded by two independent reviewers (S.S. and K.N.) based on the risk of bias, inconsistency (heterogeneity), indirectness, and imprecision, according to the GRADE guidelines (27). Each outcome was rated as high, moderate, low, or very low (**Table 4**).

### Data synthesis and meta-analysis

Mean changes and SDs for the outcomes (BMI, FPG, OGTT, HbA1c, fasting insulin, HOMA-IR, TG, TC, LDL-C, HDL-C, CRP, and MDA) in the intervention and placebo groups were used to calculate the effect sizes. For trials in which mean changes were not reported, we calculated the mean changes by considering all changes in each variable throughout the trial. We also converted 95% CIs, SEs, and IQRs to SDs applying appropriate formulas (28). Heterogeneity was determined by the *I*^2^ statistic and Cochrane's Q test. *I*^2^-value > 50% or *P* < 0.05 for the *Q*-test was characterized as significant between-study heterogeneity (29, 30). We used a random-effects model that considers the study variations to determine the overall effect sizes. To find probable sources of heterogeneity, subgroup analyses were performed according to the predefined criteria including gender (male/female), length of follow-up (8 ≥/8 < weeks), and baseline levels of outcome variables (abnormal/normal levels) and participants' baseline BMI level (normal, overweight or obese). To determine the non-linear potential effects of *Nigella sativa* dosage (g/day) on each indices, fractional polynomial modeling was executed. Sensitivity analysis was used to explore the extent to which inferences might depend on a particular study. The possibility of publication bias was evaluated by the formal Egger's test. The meta-analysis was carried out using Stata (Version 14.0, Stata Corp., College Station, TX). *P*-value <0.05 was considered as statistically significant.

## Results

### Study selection

**Figure 1:**
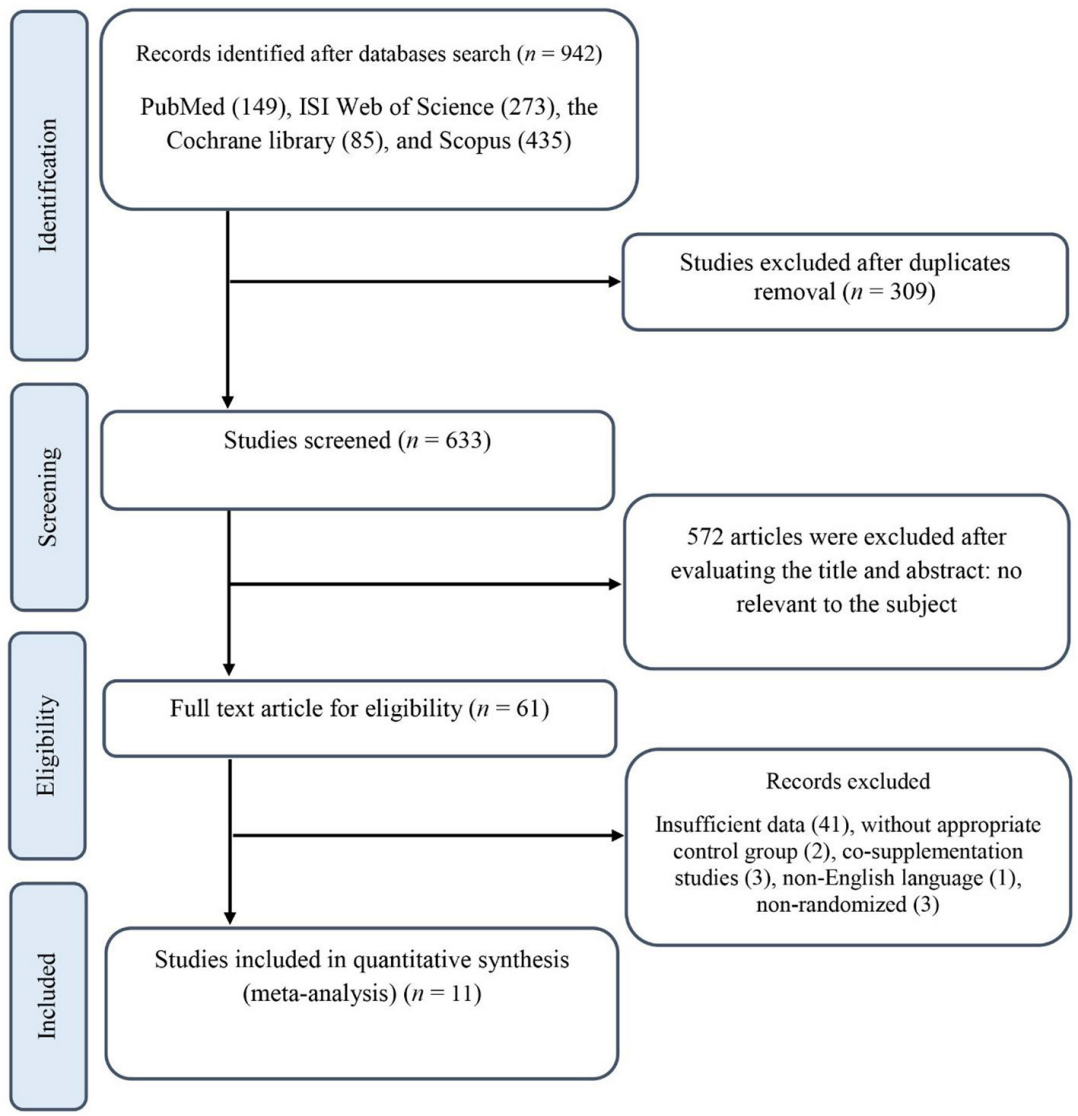
The preferred reporting items for systematic review and meta-analysis (PRISMA) flowchart.

Our initial search resulted in 942 publications, 309 of which were excluded after duplicates removal. Titles and abstracts were examined for all the remaining 633 records, resulting in the exclusion of 572 publications. Next, the full texts for 61 RCTs were checked, and 50 records were excluded for the following reasons: three studies investigated the effects of *N. sativa* combining with other supplements where the independent effect of *N. sativa* could not be distinguished (31–33); three studies had non-randomized designs (34–36); one study reported its results in a non-English language (37); two studies did not have an appropriate control group (38, 39); and the 41 remaining publications provided insufficient data and/or did not meet the inclusion criteria. The final quantitative analysis included 11 trials with 666 participants. A flow diagram of the literature search process in detail is shown in Figure 1.

### Study characteristics

The general characteristics of the 11 included studies are outlined in Table 2. All the included trials had parallel designs and were published between 2012 and 2022. These studies were carried out in Iran (19, 21, 37, 41, 43, 45, 46), India (40, 44), Egypt (20), and Saudi Arabia (42). In total, 666 participants (338 interventions and 328 controls) were recruited with the age range between 42.9 ± 3.2 and 56 ± 3.4 years old and the BMI range between 26.79 ± 2.94 and 33.90 ± 2.10 kg/m^2^. Nine studies included both genders, whereas two studies were performed exclusively on females (19, 45). All the participants were individuals with T2DM except one study including those with prediabetes (20). The dose of *N. sativa* supplementation ranged from 0.9 to 5 g/day, with the length of follow-up ranging from 2 to 6 months throughout the studies. Of the 11 RCTs, 9 effect sizes administered oil form of the supplement (20, 21, 37, 40, 41, 43–46) and the remaining used extracts of *N. sativa* (19, 42) as an investigational product. All the 11 included trials had appropriate controlled designs, and the only difference between the two groups in each study was the *N. sativa* intervention.

**Table 2:** Characteristics of the included studies.

**Studies**	**Country**	**Study design**	**Participant**	**Sex**	**Sample size**		**Trial duration (Week)**	**Means age**		**Means BMI**		**Intervention**		
					**IG**	**CG**		**IG**	**CG**	**IG**	**CG**	**NS dose (g/day)**	**NS type**	**Control group**
Najmi et al. (40)	India	Paralell, R, PC, DB	T2DM	M/F (52, 38)	40	40	8	NR	NR	30.81 ± 3.55	30.92 ± 3.67	1	NS oil extract soft gel capsules	Placebo
Hosseini et al. (41)	Iran	Paralell, R, PC, DB	T2DM	M/F (30, 40)	35	35	12	48.74 ± 7.33	50.72 ± 5.69	30.48 ± 4.00	31.83 ± 3.92	5	NS oil extract	Oil
Kaatabi et al. (42)	Saudi Arabia	Paralell, R, PC, SB	T2DM	M/F (63, 51)	57	57	12	46.82 ± 8.60	46.12 ± 6.41	29.5 ± 4.4	28.5 ± 4.3	2	NS oil extract soft gel capsules	Placebo
Heshmati et al. (43)	Iran	Paralell, R, PC, DB	T2DM	M/F (34, 38)	36	36	12	45.3 ± 6.5	47.5 ± 8.0	NR	NR	3	NS oil or sunflower soft gel capsules	Placebo
Ansari et al. (44)	India	Paralell, R, C	Diabetic nephropathy	M/F	32	31	12	53.27	48.09	28.4 ± 4.4	28.8 ± 8.1	2.5	NS oil	Conservative management of DN
Hadi et al. (45)	Iran	Paralell, R, PC, DB	T2DM	F (43)	23	20	8	51.4 ± 9.2	56.00 ± 3.4	29.01 ± 3.48	28.06 ± 4.45	1	NS oil extract soft gel capsules	Placebo
Kooshki et al. (46)	Iran	Paralell, R, PC, DB	T2DM	M/F (16, 34)	27	23	8	52.30 ± 9.43	55.91 ± 8.98	28.4 ± 4.4	28.8 ± 8.1	1	NS oil soft gel capsules	Placebo
Hadi et al. (37)	Iran	Paralell, R, PC, DB	T2DM	M/F (20, 23)	23	20	8	51.4 ± 9.2	56.00 ± 3.4	33.90 ± 2.10	33.80 ± 2.20	1	NS oil extract soft gel capsules	Placebo
Mostafa et al. (20)	Egypt	Paralell, R, PC	Prediabetic obese	M/F (16, 54)	35	35	24	44.26 ± 7.98	44.85 ± 6.19	27.64 ± 4.01	27.69 ± 1.44	0.9	NS oil soft gel capsules	Controlled diet and exercise regimen
Jangjo-Borazjani et al. (19)	Iran	Paralell, R, PC, DB	T2DM	F (20)	10	10	8	44.2 ± 4	42.9 ± 3.2	27.51 ± 4.03	26.79 ± 2.94	2	NS oil soft gel capsules	Placebo
Rahmani et al. (21)	Iran	Paralell, R, PC, DB	Diabetic hemodialysis	M/F (23, 18)	20	21	12	49.6 ± 8.75	48.57 ± 10.47	30.81 ± 3.55	30.92 ± 3.67	2	NS oil soft gel capsules	Placebo

*BMI, body mass index; IG, intervention group; CG, control group; R, randomized; PC, placebo controlled; DB, double blind; T2DM, type 2 diabetes mellitus; M, male; F, female; NR, not reported; NS, Nigella Sativa*.

### Effects of *N. sativa* supplementation on cardiometabolic parameters

#### Anthropometric measurement

A total of five studies investigated the effects of *N. sativa* on BMI values (19, 20, 37, 40, 43). Pooled results from the random-effects model indicated that BMI values did not change significantly after *N. sativa* supplementation (WMD: −0.56 kg/m^2^; 95% CI: −1.95, 0.81; *P* = 0.422; *P*_heterogeneity_ < 0.001, *I*^2^ = 83.2%) (Supplementary Figure 1). Subgroup analysis demonstrated that although *N. sativa* had a significant lowering effect on BMI in studies with the supplemented dose of more than 1 g/day, this effect was irrespective of the health condition of the participants (Table 3).

**Table 3:** Subgroup analyses of NS intake on body mass index, glycemic indices, lipid profile, CRP, and MDA.

	**Number of studies**	**WMD (95%CI)**	***P*-value**	**Heterogeneity**	
				***P*_heterogeneity_**	***I* ^2^**
**Subgroup analyses of NS intake on BMI (kg/m** ^2^ **)**					
Overall effect	5	−0.56 (−1.95, 0.81)	0.422	<0.001	83.2%
**Baseline BMI (kg/m** ^2^ **)**					
Overweight (25–29.9)	3	−0.97 (−2.20, 0.25)	0.120	0.249	28.1%
Obese (>30)	2	−0.20 (−2.64, 2.23)	0.868	<0.001	93.4%
**Trial duration (week)**					
≤ 8	2	−0.45 (−2.42, 1.51)	0.652	0.209	36.5%
>8	3	−0.61 (−2.47, 1.25)	0.521	<0.001	90.9%
**NS dose (g/day)**					
≤ 1	2	−0.01 (−2.50, 2.46)	0.988	0.045	75.1%
>1	3	−1.12 (−2.10, −0.15)	0.023	0.218	34.3%
**Health status**					
T2DM	4	−1.24 (−1.98, −0.49)	0.001	0.372	4.1%
Prediabetic	1	1.00 (0.35, 1.64)	0.003	-	-
**Subgroup analyses of NS intake on FPG (mg/dL)**					
Overall effect	10	−24.18 (−39.36, −9.00)	0.002	<0.001	98.7%
**Trial duration (week)**					
≤ 8	4	−29.50 (−61.75, 2.74)	0.073	<0.001	95.5%
>8	6	−21.26 (−40.83, −1.68)	0.033	<0.001	99.2%
**NS dose (g/day)**					
≤ 1	4	−29.90 (−55.87, −3.94)	0.024	<0.001	96.7%
>1	6	−20.96 (−33.33, −8.60)	0.001	<0.001	92.8%
**Health status**					
T2DM	9	−26.65 (−38.24, −15.06)	<0.001	<0.001	93.5%
Prediabetic	1	0.60 (−1.50, 2.70)	0.576	-	-
**Subgroup analyses of NS intake on HbA1c (%)**					
Overall effect	7	−0.54 (−0.82, −0.26)	<0.001	<0.001	94.4%
**Trial duration (week)**					
≤ 8	2	−1.00 (−1.89, −0.11)	0.027	0.004	87.9%
>8	5	−0.41 (−0.71, −0.11)	0.007	<0.001	94.7%
**NS dose (g/day)**					
≤ 1	3	−0.62 (−1.25, −0.00)	0.050	<0.001	95.9%
>1	4	−0.51 (−0.74, −0.28)	<0.001	0.001	80.5%
**Health status**					
T2DM	6	−0.61 (−0.83, −0.39)	<0.001	<0.001	82.0%
Prediabetic	1	0.00 (−0.07, 0.07)	1.000	-	-
**Subgroup analyses of NS intake on OGTT (mg/dL)**					
Overall effect	4	−12.28 (−29.94, 5.38)	0.173	<0.001	90.7%
**Subgroup analyses of NS intake on fasting insulin (**μ**IU/ml)**					
Overall effect	5	1.06 (−1.87, 4.00)	0.477	<0.001	95.8%
**Trial duration (week)**					
≤ 8	2	4.27 (−2.10, 10.65)	0.189	0.001	91.2%
>8	3	−0.80 (−4.64, 3.04)	0.682	<0.001	97.4%
**NS dose (g/day)**					
≤ 1	2	3.00 (−5.90, 11.90)	0.509	<0.001	96.3%
>1	3	0.07 (−3.79, 3.93)	0.971	<0.001	94.1%
**Health status**					
T2DM	4	1.75 (−1.79, 5.31)	0.332	<0.001	93.1%
Prediabetic	1	−1.39 (−2.14, −0.64)	<0.001	-	-
**Subgroup analyses of NS intake on HOMA-IR**					
Overall effect	5	−0.20 (−0.92, 0.50)	0.572	<0.001	88.6%
**Trial duration (week)**					
≤ 8	2	1.32 (−0.61, 3.26)	0.181	0.009	85.4%
>8	3	−0.80 (−1.34, −0.27)	0.003	0.009	78.6%
**NS dose (g/day)**					
≤ 1	2	0.85 (−2.02, 3.73)	0.559	<0.001	94.6%
>1	3	−0.68 (−1.78, 0.41)	0.222	<0.001	87.8%
**Health status**					
T2DM	4	−0.03 (−1.30, 1.23)	0.957	<0.001	91.2%
Prediabetic	1	−0.54 (−0.79, −0.28)	<0.001	-	-
**Subgroup analyses of NS intake on TG (mg/dL)**					
Overall effect	7	−11.85 (−29.83, 6.12)	0.196	<0.001	90.1%
**Baseline TG (mg/dL)**					
<150	2	−1.32 (−5.00, 2.36)	0.482	0.547	0.0%
≥150	5	−18.64 (−51.34, 14.05)	0.264	<0.001	93.1%
**Trial duration (week)**					
≤ 8	4	−14.06 (−59.09, 30.97)	0.541	<0.001	94.6%
>8	3	−8.91 (−21.22, 3.40)	0.156	0.073	61.9%
**NS dose (g/day)**					
≤ 1	4	−14.72 (−45.38, 15.92)	0.346	<0.001	94.5%
>1	3	−10.76 (−24.67, 3.15)	0.130	0.226	32.9%
**Health status**					
T2DM	6	−14.57 (−42.34, 13.19)	0.304	<0.001	91.6%
Prediabetic	1	−1.50 (−5.22, 2.22)	0.430	-	-
**Subgroup analyses of NS intake on TC (mg/dL)**					
Overall effect	6	−23.84 (−39.25, −8.44)	0.002	<0.001	91.9%
**Baseline TC (mg/dL)**					
<200	3	−14.35 (−32.02, 3.31)	0.111	<0.001	87.2%
≥200	3	−33.59 (−58.26, −8.92)	0.008	<0.001	89.6%
**Trial duration (week)**					
≤ 8	3	−34.35 (−57.05, −11.64)	0.003	0.002	84.1%
>8	3	−13.73 (−28.89, 1.42)	0.076	<0.001	88.5%
**NS dose (g/day)**					
≤ 1	3	−25.83 (−59.66, 7.98)	0.134	<0.001	95.3%
>1	3	−21.80 (−30.70, −12.91)	<0.001	0.203	37.2%
**Health status**					
T2DM	5	−28.74 (−42.55, −14.93)	<0.001	0.001	79.8%
Prediabetic	1	−1.30 (−5.29, 2.69)	0.523	-	-
**Subgroup analyses of NS intake on LDL (mg/dL)**					
Overall effect	7	−20.12 (−33.72, −6.51)	0.004	<0.001	92.6%
**Baseline LDL (mg/dL)**					
<130	4	−16.70 (−33.32, −0.08)	0.049	<0.001	89.3%
≥130	3	−24.51 (−42.97, −6.05)	0.009	<0.001	88.7%
**Trial duration (week)**					
≤ 8	4	−28.21 (−35.93, −20.48)	<0.001	0.146	44.2%
>8	3	−11.95 (−29.61, 5.71)	0.185	89.1%	
**NS dose (g/day)**					
≤ 1	4	−18.37 (−38.89, 2.14)	0.079	<0.001	95.3%
>1	3	−22.48 (−39.53, −5.44)	0.010	0.003	82.5%
**Health status**					
T2DM	6	−24.13 (−33.81, −14.45)	<0.001	0.001	74.9%
Prediabetic	1	−0.40 (−5.35, 4.55)	0.874	-	-
**Subgroup analyses of NS intake on HDL (mg/dL)**					
Overall effect	7	0.56 (−1.98, 3.11)	0.663	<0.001	87.3%
**Baseline HDL (mg/dL)**					
<40	1	6.10 (4.27, 7.92)	<0.001	-	-
≥40	6	−0.53 (−1.84, 0.77)	0.421	0.165	36.2%
**Trial duration (week)**					
≤ 8	4	−0.23 (−2.51, 2.04)	0.840	0.061	59.2%
>8	3	1.41 (−3.44, 6.28)	0.568	<0.001	94.1%
**NS dose (g/day)**					
≤ 1	4	−0.79 (−2.06, 0.47)	0.220	0.301	18.0%
>1	3	2.63 (−2.19, 7.46)	0.285	<0.001	89.8%
**Health status**					
T2DM	6	0.77 (−2.32, 3.86)	0.626	<0.001	88.3%
Prediabetic	1	−0.60 (−2.30, 1.10)	0.490	-	-
**Subgroup analyses of NS intake on CRP (mg/L)**					
Overall effect	3	−1.05 (−1.75, −0.35)	0.003	0.001	85.4%
**Subgroup analyses of NS intake on MDA (**μ**mol/l)**					
Overall effect	3	−1.27 (−2.53, −0.01)	0.048	<0.001	90.3%

BMI, body mass index; FPG, fasting plasma glucose; HbA1c, hemoglobin A1c; OGTT, oral glucose tolerance test; HOMA-IR, homeostatic model assessment of insulin resistance; TG, triglyceride; TC, total cholesterol; LDL-C, low-density lipoprotein cholesterol; HDL-C, high-density lipoprotein cholesterol; CRP, C-reactive protein; MDA, Malondialdehyde.

### Measures of glucose homeostasis

#### Glycemic control

**Figure 2:**
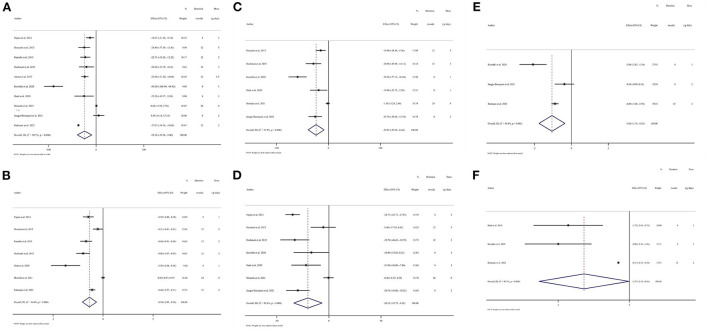
Forest plots of randomized controlled trials illustrating weighted mean difference (WMD) in biomarkers between the intervention and placebo groups for all eligible studies in overall analysis. **(A)** Fasting plasma glucose (FPG); **(B)** hemoglobin A1c (HbA1c); **(C)** total cholesterol (TC); **(D)** low-density lipoprotein cholesterol (LDL-C); **(E)** C-reactive protein (CRP); **(F)** Malondialdehyde (MDA).

Results from the random-effects model indicated that consumption of *N. sativa* resulted in a significant reduction in FPG (WMD: −24.18 mg/dL; 95% CI: −39.36, −9; *P* = 0.002; *P*_heterogeneity_ < 0.001, *I*^2^ = 98.7%) (19–21, 37, 40–44, 46), and HbA1c (WMD: −0.54%; 95% CI: −0.82, −0.26; *P* < 0.001; *P*_heterogeneity_ < 0.001, *I*^2^ = 94.4%) (20, 21, 37, 40–43) (Figure 2). However, there was no significant difference in OGTT (WMD: −12.28 mg/dL; 95% CI: −29.94, 5.38; *P* = 0.173; *P*_heterogeneity_ < 0.001, *I*^2^ = 90.7%) between groups (40–42, 44) (Supplementary Figure 1). The findings from the subgroup analyses showed that *N. sativa* reduced FPG in those with T2DM and individuals who were intervened with *N. sativa* for a longer time (more than 8 weeks) regardless of the dose of intervention. Also, a significant decrease in HbA1c was observed in subjects with T2DM irrespective of both the length of follow-up and the intervention dose of *N. sativa*. Subgroup analysis was not conducted on OGTT, as there were not enough studies reporting on this parameter (Table 3).

### Insulin resistance and secretion

The meta-analysis of five trials revealed no significant change in fasting insulin levels (WMD: 1.06 μIU/mL; 95% CI: −1.87, 4; *P* = 0.477; *P*_heterogeneity_ < 0.001, *I*^2^ = 95.8%) (19–21, 37, 43) and in HOMA-IR (WMD: −0.20; 95% CI: −0.92, 0.50; *P* = 0.572; *P*_heterogeneity_ < 0.001, *I*^2^ = 88.6%) (19, 20, 37, 42, 43) after *N. sativa* intervention (Supplementary Figure 1). On subgroup analyses, we observed that *N. sativa* supplementation significantly reduced fasting insulin concentration among subjects with prediabetes. Furthermore, studies with a longer intervention duration (more than 8 weeks) of *N. sativa* supplementation and those including individuals with prediabetes reported a significant reduction in serum HOMA-IR. Notably, no evidence of difference was shown in HOMA-IR based on the dose of intervention (Table 3).

### Cardiovascular risk factors

#### Lipid profile

Pooled data from six studies indicated that TC levels were reduced significantly in those receiving *N. sativa* compared to controls (WMD: −23.84 mg/dL; 95% CI: −39.25, −8.44; *P* = 0.002; *P*_heterogeneity_ < 0.001, *I*^2^ = 91.9%) (19, 20, 37, 41, 43, 46) (Figure 2). Moreover, the effect of *N. sativa* supplementation on LDL-C was evaluated in seven clinical trials and the pooled mean difference revealed a reduction in LDL-C (WMD: −20.12 mg/dL; 95% CI: −33.72, −6.51; *P* = 0.004; *P*_heterogeneity_ < 0.001, *I*^2^ = 92.6%) (19, 20, 37, 40, 41, 43, 46) (Figure 2). However, combining seven effect sizes revealed that *N. sativa* supplementation resulted in a non-significant change in serum levels of TG (WMD: −11.85 mg/dL; 95% CI: −29.83, 6.12; *P* = 0.196; *P*_heterogeneity_ < 0.001, *I*^2^ = 90.1%) (19, 20, 37, 40, 41, 43, 46) (Supplementary Figure 1) and HDL-C (WMD: 0.56 mg/dL; 95% CI: −1.98, 3.11; *P* = 0.663; *P*_heterogeneity_ < 0.001, *I*^2^ = 87.3%) (19, 20, 37, 40, 41, 43, 46) (Supplementary Figure 1). The subgroup analyses indicated that *N. sativa* consumption might induce a more significant reduction of TC and LDL-C concentrations compared to the placebo group in those with T2DM, individuals with more than 1 g/day of *N. sativa* supplementation, and the duration of follow-up was 8 weeks or less. Additionally, reduced TC levels were significant in subjects with a baseline TC level of 200 mg/dL or more. However, LDL-C changes were not associated with the baseline levels of LDL-C. Results for TG remained non-significant across all subgroups, while after *N. sativa* consumption, HDL-C levels were significantly elevated in those with baseline serum levels of HDL-C lower than 40 mg/dL (Table 3).

### Inflammatory and oxidative stress markers

Pooling three effect sizes, a significant reduction was seen in CRP (WMD: −1.05 mg/L; 95% CI: −1.75, −0.35; *P* = 0.003; *P*_heterogeneity_ = 0.001, *I*^2^ = 85.4%) and MDA (WMD: −1.27 μmol/L; 95% CI: −2.53, −0.01; *P* = 0.048; *P*_heterogeneity_ < 0.001, *I*^2^ = 90.3%) following *N. sativa* supplementation (Figure 2). However, it was impossible to perform subgroup analysis for CRP and MDA due to the limited number of included studies reporting these parameters (Table 3).

### Sensitivity analysis

Sensitivity analysis for BMI, OGTT, and TG showed that the overall estimates were influenced by eliminating studies conducted by Mostafa et al. (20) (WMD: −1.24; 95% CI: −1.98, −0.49), Hadi et al. (37) (WMD: −0.57; 95% CI: −1.13, −0.01), and Najmi et al. (40) (WMD: −19.27; 95% CI: −37.44, −1.97), respectively. Moreover, the exclusion of the study conducted by Rahmani et al. (21) (WMD: −1.2; 95% CI: −2.87, 0.45) changed the overall effect size for CRP. Furthermore, the results of sensitivity analysis for MDA showed that removing the Hadi et al. (37) (WMD: −1.08; 95% CI: −2.73, 0.56) and Kooshki et al. (46) (WMD: −0.92; 95% CI: −2.29, 0.44) studies changed the overall effect sizes. Finally, sensitivity analysis for FPG, HbA1c, fasting insulin, HOMA-IR, LDL-C, and HDL-C did not indicate any sensitivity.

### Publication bias

Funnel plot (Supplementary Figure 2), Egger's test (47), and Begg's test (48) (Supplementary Table 3) were applied to assess the possibility of significant publication bias. The findings from Egger's statistical test revealed that no evidence of publication bias was detected for BMI, FPG, fasting insulin, HOMA-IR, TG, LDL-C, HDL-C, CRP, or MDA. Visual inspection of funnel plot also affirmed these findings on BMI, FPG, fasting insulin, HOMA-IR, TG, LDL-C, and HDL-C. However, there were significant publication bias for the studies investigating the effectiveness of *N. sativa* on OGTT (*P* = 0.006), HbA1c (*P* = 0.005), and TC (*P* = 0.01).

### GRADE assessment

The certainty of the evidence was evaluated by applying the GRADE protocol (Table 4). It was determined that the quality of evidence for FPG, LDL-C, CRP, and MDA was low due to very serious inconsistency (*I*^2^ > 75%). However, for BMI, OGTT, fasting insulin, HOMA-IR, TG, and HDL-C, the quality of evidence was downgraded to very low owing to very serious inconsistency (*I*^2^ > 75%) and imprecision (Wide CI). In addition, the quality of evidence for HbA1c and TC was also very low due to very serious inconsistency (*I*^2^ > 75%) and significant publication bias.

**Table 4:** GRADE profile of NS intake on lipid profile, glycemic indices, body mass index, CRP, and MDA.

**Outcomes**	**Risk of bias**	**Inconsistency**	**Indirectness**	**Imprecision**	**Publication bias**	**Number of intervention/** **control**	**Quality of evidence**
BMI	No serious limitation	Very serious limitation^a^	No serious limitation	Serious limitation^b^	No serious limitation	275 (139/136)	⊕○○○^d^ Very low
FPG	No serious limitation	Very serious limitation^a^	No serious limitation	No serious limitation	No serious limitation	623 (315/308)	⊕⊕○○ Low
HbA1c	No serious limitation	Very serious limitation^a^	No serious limitation	No serious limitation	Serious limitation^c^	490 (246/244)	⊕○○○ Very low
OGTT	No serious limitation	Very serious limitation^a^	No serious limitation	Serious limitation^b^	Serious limitation^c^	283 (142/141)	⊕○○○ Very low
Fasting insulin	No serious limitation	Very serious limitation^a^	No serious limitation	Serious limitation^b^	No serious limitation	246 (124/122)	⊕○○○ Very low
HOMA-IR	No serious limitation	Very serious limitation^a^	No serious limitation	Serious limitation^b^	No serious limitation	319 (161/158)	⊕○○○ Very low
TG	No serious limitation	Very serious limitation^a^	No serious limitation	Serious limitation^b^	No serious limitation	405 (206/199)	⊕○○○ Very low
TC	No serious limitation	Very serious limitation^a^	No serious limitation	No serious limitation	Serious limitation^c^	325 (166/159)	⊕○○○ Very low
LDL	No serious limitation	Very serious limitation^a^	No serious limitation	No serious limitation	No serious limitation	325 (166/159)	⊕⊕○○ Low
HDL	No serious limitation	Very serious limitation^a^	No serious limitation	Serious limitation^b^	No serious limitation	325 (166/159)	⊕○○○ Very low
CRP	No serious limitation	Very serious limitation^a^	No serious limitation	No serious limitation	No serious limitation	111 (57/54)	⊕⊕○○ Low
MDA	No serious limitation	Very serious limitation^a^	No serious limitation	No serious limitation	No serious limitation	274 (140/134)	⊕⊕○○ Low

^a^There is significant heterogeneity for BMI (I^2^ = 83.2%), FPG (I^2^ = 74.3%), HbA1c (I^2^ = 87.6%), OGTT (I^2^ = 90.7%), fasting insulin (I^2^ = 64.3%), HOMA-IR (I^2^ = 59.1%), TG (I^2^ = 90.1%), TC (I^2^ = 91.9%), LDL-C (I^2^ = 92.6%), HDL-C (I^2^ = 87.3%), CRP (I^2^ = 85.4%), MDA (I^2^ = 90.3%).

^b^There is no evidence of significant effects (95%CI includes zero).

^c^There is significant publication bias.

^d^⊕ = serious limitation; ○ = no serious limitation.

BMI, body mass index; FPG, fasting plasma glucose; HbA1c, hemoglobin A1c; OGTT, oral glucose tolerance test; HOMA-IR, homeostatic model assessment of insulin resistance; TG, triglyceride; TC, total cholesterol; LDL-C, low-density lipoprotein cholesterol; HDL-C, high-density lipoprotein cholesterol; CRP, C-reactive protein; MDA, Malondialdehyde.

## Discussion

Many functional plants and their bioactive components have been demonstrated to possess anti-diabetic effects, such as tea (49), sweet tea (50), ginseng (29), citrus (51), pomegranate peel (52), and some medicinal plants (53). The present meta-analysis investigated the effectiveness of *N. sativa* consumption on cardiometabolic indicators among individuals with prediabetes and T2DM. The result indicated that *N. sativa* supplementation was associated with declines in glycemic control components (FPG and HbA1c), lipid profile parameters (TC and LDL-C), and biomarkers of inflammation and oxidative stress (CRP and MDA). Meanwhile, it was found that *N. sativa* supplementation enhanced serum levels of HDL-C in subjects with the baseline HDL-C level lower than 40 mg/dL. Furthermore, HOMA-IR and BMI values decreased in the *N. sativa*-supplemented group compared to the control group, when the length of follow-up was more than 8 weeks and in individuals with more than 1 g/day of *N. sativa* supplementation, respectively. However, there was no change in fasting serum concentration of insulin, TG, or glucose values following OGTT after *N. sativa* supplementation compared to the control group.

*Nigella sativa* contains various phytochemicals, including thymoquinone (TQ) which is the main ingredient of this plant. Moreover, thymol and dithymoquinone are other components of *N. sativa* (54). Current evidence proposes that *N. sativa* intake contributes to the modulation of cardiometabolic parameters in diabetes through the improvement of glucose homeostasis and lipid profiles, resulting in the prevention of atherosclerosis and cardiovascular events as diabetes complications (55). In 2017, a systematic review and meta-analysis of seven RCTs demonstrated that intake of *N. sativa* improves glycemic control parameters by reducing serum levels of FPG and HbA1c, and improving lipid profile through decreasing TC and LDL-C concentrations, without any significant alterations in TG or HDL-C (56). This is in line with the present study, which reveals that *N. sativa* favorably affects glycemic and lipid profiles. Moreover, our results revealed that *N. sativa* supplementation might modulate inflammation and oxidative stress by decreasing CRP and MDA levels.

Our findings are also supported by the previous systematic reviews and meta-analyses demonstrating the hypoglycemic efficacy of *N. sativa* among various non-diabetic populations (57–59). Hallajzadeh et al. (58) reported the beneficial effects of *N. sativa* on FPG and HbA1c without any change in serum insulin levels. Moreover, *N*. *sativa* consumption had a significant lowering effect on FPG, HbA1c, and OGTT values in a study conducted by Askari et al. (57). *N. sativa* could preserve pancreatic β-cell integrity, enhance the quantity of islets, and consequently make a contribution to the pancreatic β-cells proliferation and decreasing insulin resistance in diabetic experimental models and clinical studies (38, 43, 60, 61). Other probable anti-hyperglycemic mechanisms of *N. sativa* are as follows. Thymoquinone, a main bioactive compound in *N. sativa*, may reduce the gene expression of fructose-1,6-bisphosphatase and glucose-6-phosphatase, resulting in diminishing the hepatic gluconeogenesis (62, 63). *N. sativa* may also exert its hypoglycemic effects through activation of the AMP-activated protein kinase (AMPK) pathway, resulting in enhancement of pancreatic insulin secretion (22, 62). *N. sativa* may reduce glucose absorption by inhibiting the sodium glucose co-transporter (64). Similarly, it was reported that *N. sativa* could decrease intestinal glucose absorption and increase tissue glucose uptake by improving insulin release (42, 65). It is noteworthy that in the present study, we did not find any favorable effects of *N. sativa* supplementation on fasting serum levels of insulin and insulin resistance. This discrepancy might be partly due to the limited number of included studies and different forms of *N. sativa* supplementation. Therefore, further studies are warranted in order to verify the effectiveness of *N. sativa* on insulin secretion and/or insulin resistance.

Insulin resistance precedes the development of T2DM, leading to increased levels of free fatty acids (FFAs), and gives rise to TG formation, which in turn results in developing dyslipidemia among subjects with T2DM (66–68). Lipid-lowering property of *N. sativa* has been proposed to be attributed to its bioactive ingredients. Polyunsaturated fatty acids (PUFAs) (e.g., linoleic acid) in *N. sativa* were reported to inhibit the secretion of very-low density lipoprotein cholesterol (VLDL-C) and enhance fatty acid oxidation (69). Its high content of soluble fibers could contribute to reducing cholesterol absorption and elevating bile production (36). Furthermore, its TQ was reported to suppress gene expression of 3-hydroxy-3-methylglutaryl coenzyme A reductase (HMG-COAR), up-regulate insulin receptors in hepatic cells, and enhance the uptake of LDL-C (70). A recent meta-analysis among the general population revealed a significant lowering effect of *N. sativa* on TC and LDL-C concentrations, while no effect on TG or HDL-C levels (59). These findings are in total agreement with the present study. However, another systematic review and meta-analysis among healthy and unhealthy participants demonstrated its efficacy on serum levels of TG in addition to TC and LDL-C concentrations (58). This discrepancy might be caused by the different target populations and the number of the included studies.

Low-grade chronic inflammation and oxidative stress seem to be independent risk factors for the development of T2DM (70, 71). One of the well-established indicators of inflammation in individuals with T2DM is CRP (72). The current finding was affirmed by a recent systematic review and meta-analysis, which reported that elevated CRP concentrations were linked to the increased incidence of T2DM (73). Moreover, it is also notable that studies showed that MDA, as a toxic by-product of oxidation, increases T2DM pathogenesis (74). Emerging evidence supports the beneficial effects of *N. sativa* on indicators of inflammation through inhibition of the nuclear factor-kappa B (NF-κB) pathway and oxidative stress *via* increasing the expression of antioxidant enzymes, such as superoxide dismutase, in T2DM (75). However, previous systematic reviews and meta-analyses did not show this favorable effect (58, 76). These findings are in contrast to the present study, which demonstrated that *N. sativa* intervention could reduce CRP and MDA concentrations in individuals with prediabetes and T2DM. The possible explanation for this discrepancy might be due to the great data range and standard deviation among the included studies.

Obesity usually co-occurs with diabetes (77). Available experimental evidence proposed that *N. sativa* supplementation could exert its anti-obesity effect through appetite suppression (78, 79). A meta-analysis of 11 RCTs in the general population by Namazi et al. (80) revealed that *N. sativa* oil significantly decreased BMI values in adult humans. However, the present study failed to show its beneficial effects on BMI. This might be mainly due to the limited number of studies included in this meta-analysis reporting on the effectiveness of *N. sativa* on BMI values and the differences in the health status of the participants.

### Strengths and limitations

This is the first systematic review and meta-analysis investigating the effectiveness of *N. sativa* supplementation on a wide range of cardiometabolic indicators in individuals with prediabetes and T2DM. The previous study included 505 participants (7 papers) (56), whereas our systematic review includes 666 participants (11 papers). In addition, subgroup analyses and grading the overall certainty of evidence across the studies according to the GRADE guidelines were conducted. Despite all the aforementioned strengths, the present study has some limitations that should be taken into consideration. First, we were unable to stratify the efficacy of *N. sativa* on each endpoint based on different forms of *N. sativa* (extract vs. oil) due to the limited number of included studies. Second, the RCTs had relatively small sample sizes, with only one study including more than 114 participants. Third, all the studies were carried out in Asia, except one of them performed in Africa, which may lead to generalizability limitations. Fourth, in our analysis, statistical heterogeneity is evident. Poor methodological quality and/or differences in treatment regimens (doses/durations) or the form of *N. sativa* used may contribute to these differences. Finally, we did not consider the effects of some confounding factors, such as smoking status and diabetes duration, owing to the insufficient reporting of them.

## Conclusions

The results of the present study have indicated that *N. sativa* may improve cardiometabolic parameters by ameliorating glucose homeostasis and alleviating dyslipidemia, inflammation, and oxidative stress in individuals with prediabetes and T2DM. Overall, our results suggest that *N. sativa* supplementation can be a potential adjuvant therapy in the management of prediabetes and T2DM. In the future, more well-designed clinical studies are guaranteed to shed light on these findings.

## Author contributions

SS and KN contributed to the literature search and data extraction. SS, KN, and OA contributed to data analysis. SS, KN, and KA drafted the manuscript and which was critically revised for important intellectual content by all authors. SS, KN, PZ, and H-BL contributed to the methodology, statistical analysis, and manuscript drafting. R-YG supervised the study, is a guarantor and had full access to all the data and takes responsibility for the integrity of the data, and the accuracy of the data analysis. All authors have read and agreed to the published version of the manuscript.

## Funding

This study was funded by the Agricultural Science and Technology Innovation Program (ASTIP-IUA-2022002).

## Conflict of interest

The authors declare that the research was conducted in the absence of any commercial or financial relationships that could be construed as a potential conflict of interest.

## Publisher's note

All claims expressed in this article are solely those of the authors and do not necessarily represent those of their affiliated organizations, or those of the publisher, the editors and the reviewers. Any product that may be evaluated in this article, or claim that may be made by its manufacturer, is not guaranteed or endorsed by the publisher.
