# Supplementary material for: Nigella sativa supplementation improves cardiometabolic indicators in population with prediabetes and type 2 diabetes mellitus: A systematic review and meta-analysis of randomized controlled trials
Source: Front Nutr. 2022 Aug 11;9:977756. doi: 10.3389/fnut.2022.977756 (PMC9403837; doi:10.3389/fnut.2022.977756)
Supplement: Supplementary file 1 [file Table_1.DOCX]

**Supplementary Figure 1.** Forrest plots


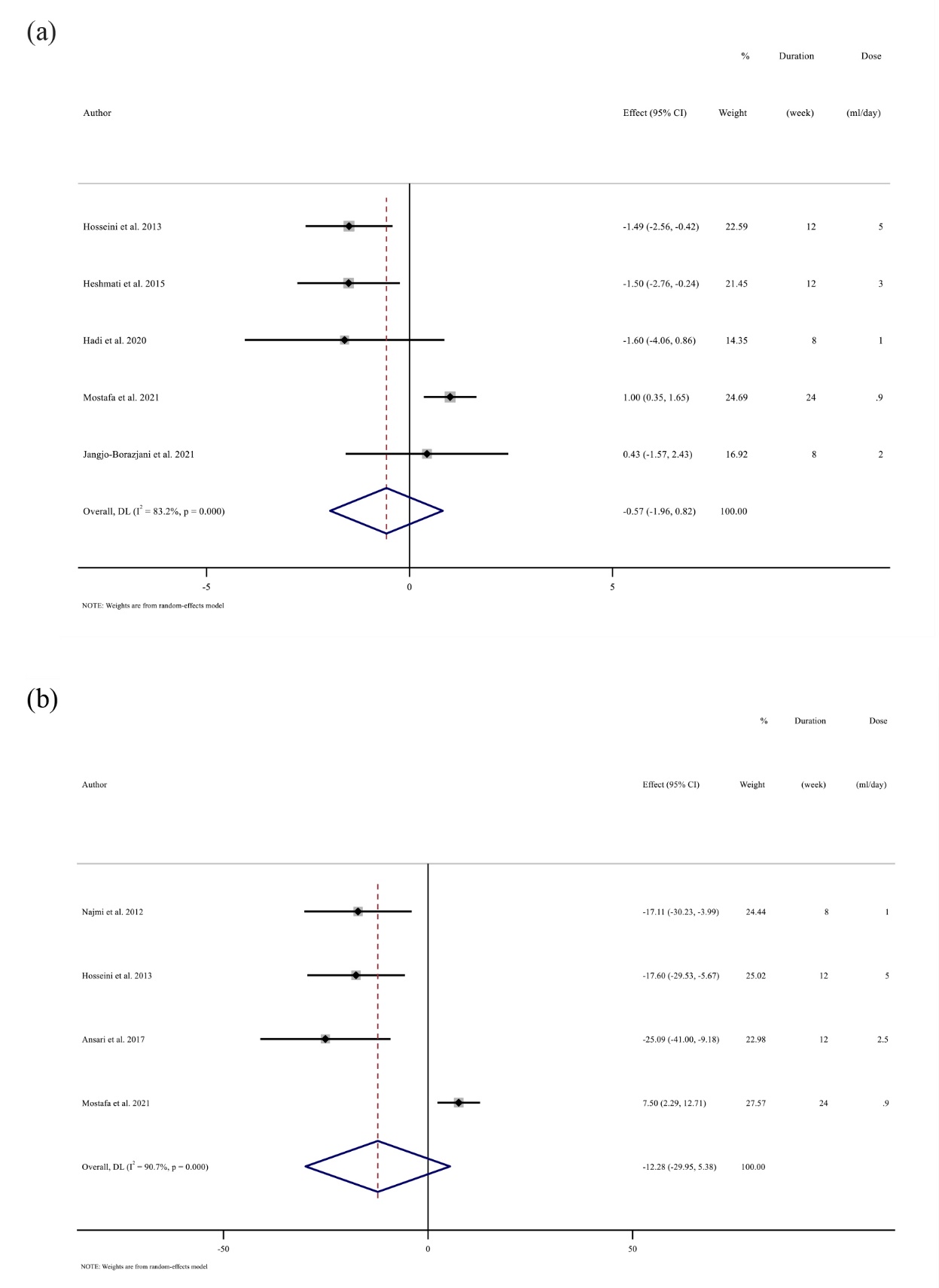


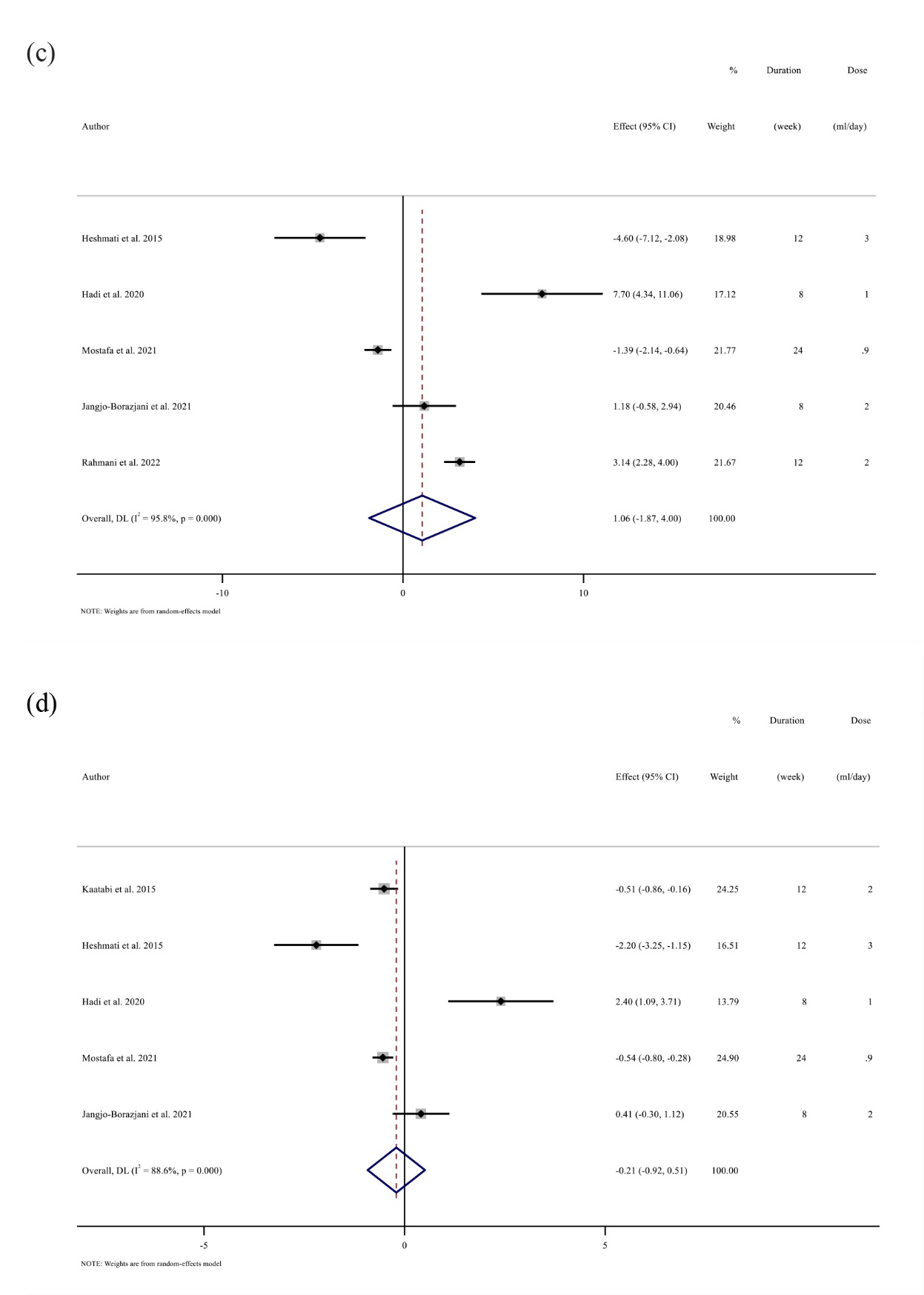


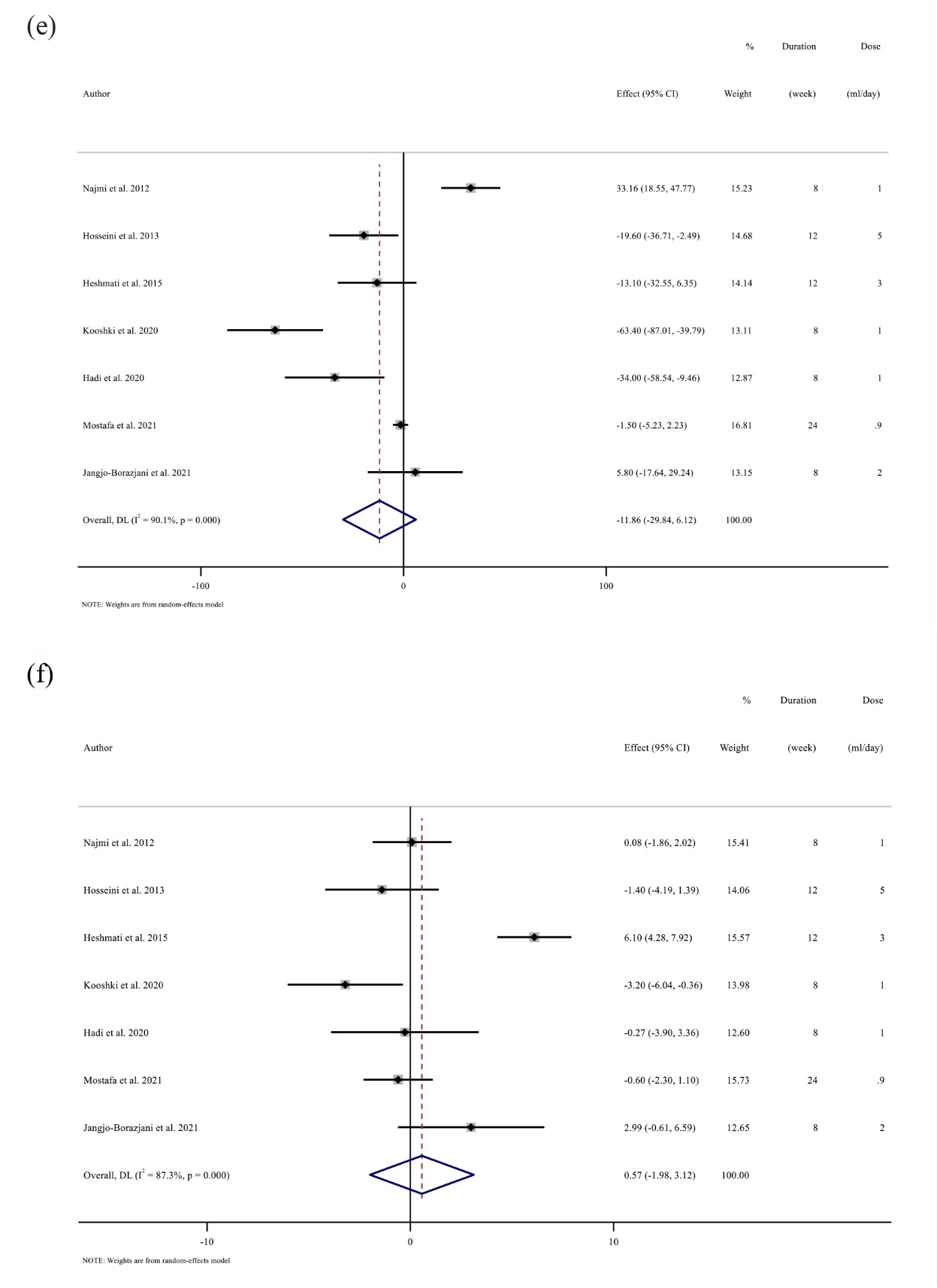


a: body mass index (BMI); b: oral glucose tolerance test (OGTT); c: fasting insulin; d: homeostatic model assessment of insulin resistance (HOMA-IR); e: triglyceride (TG); f: high-density lipoprotein cholesterol (HDL-C).

**Supplementary Figure 2.** Funnel plots


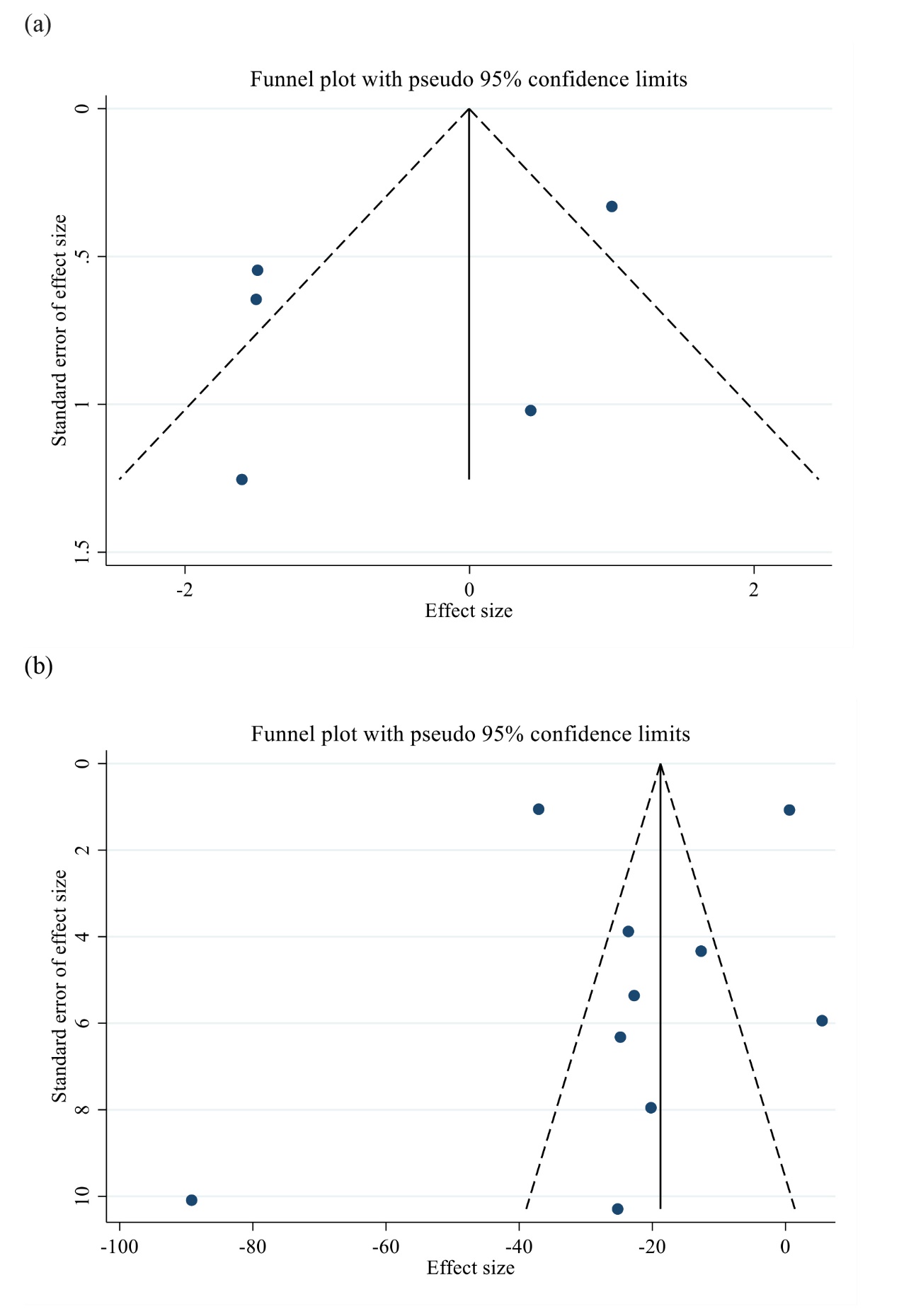


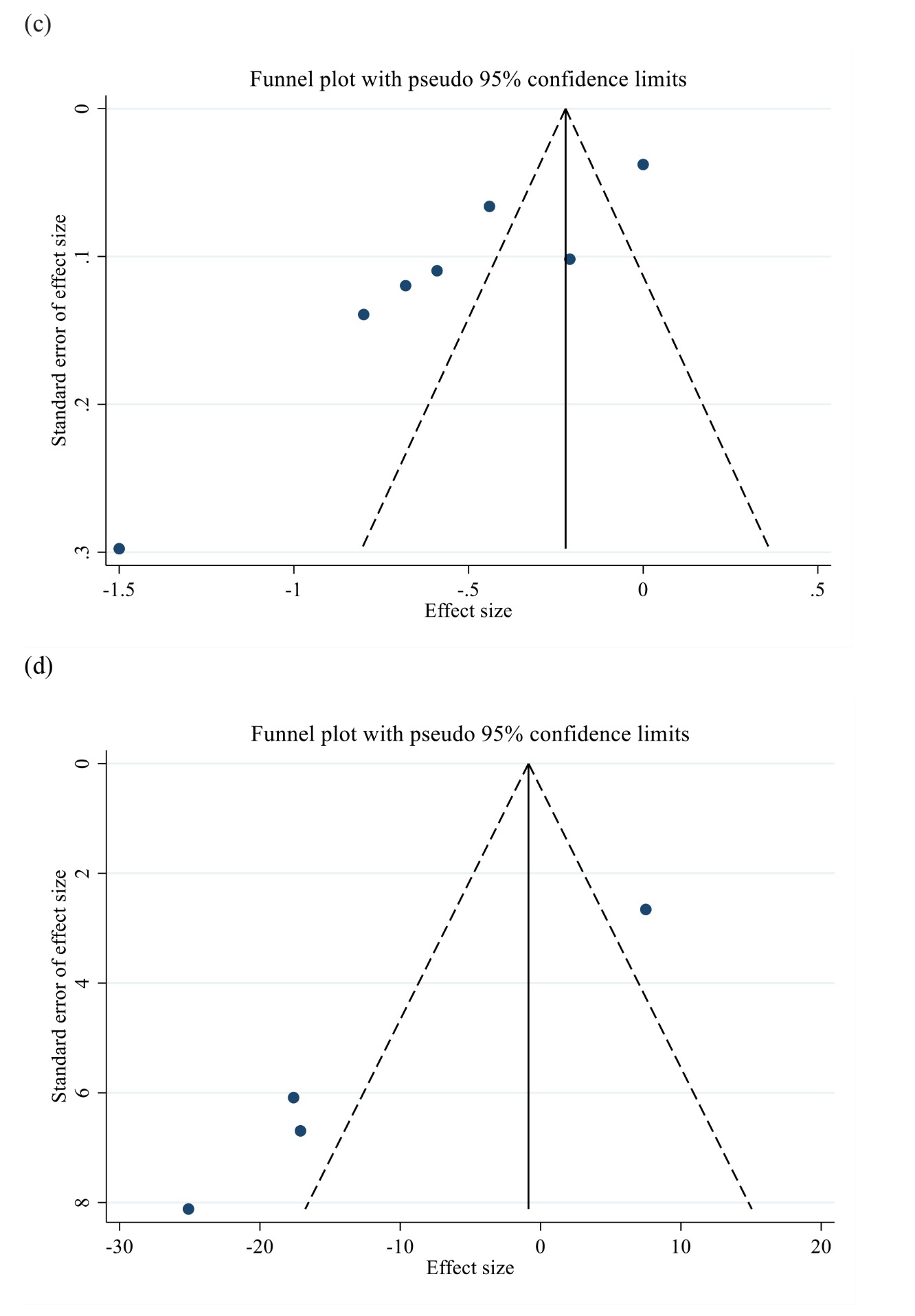


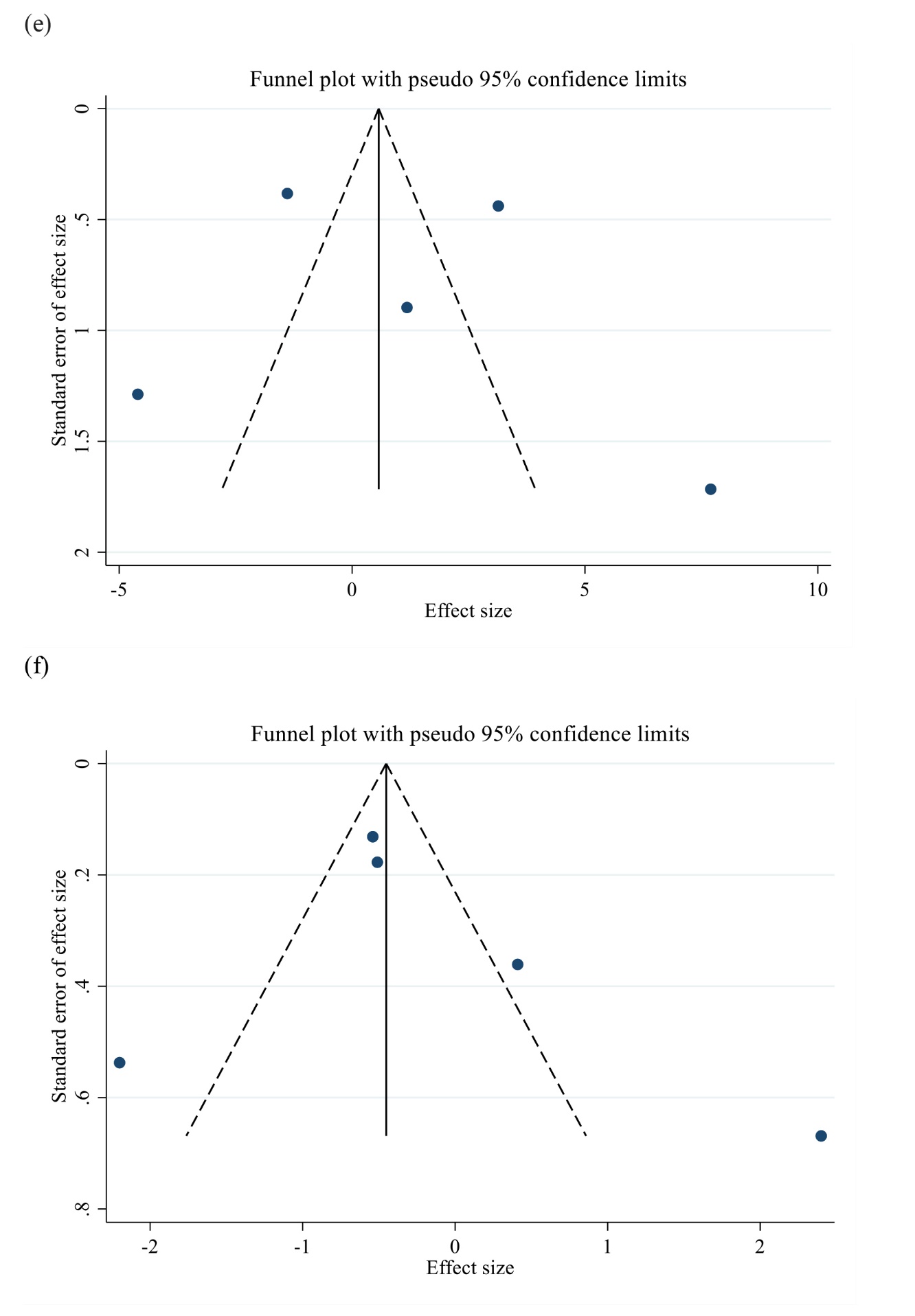


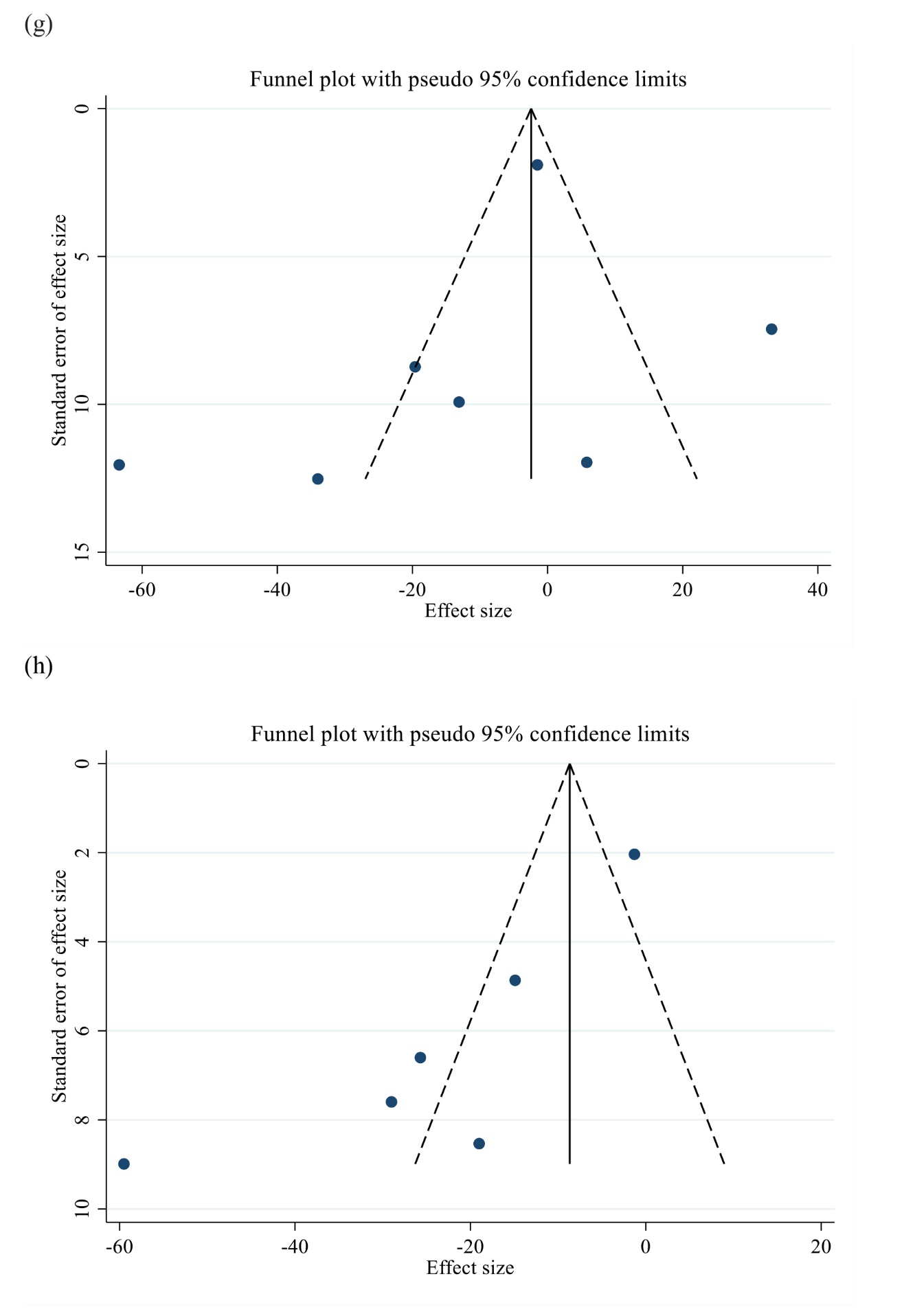


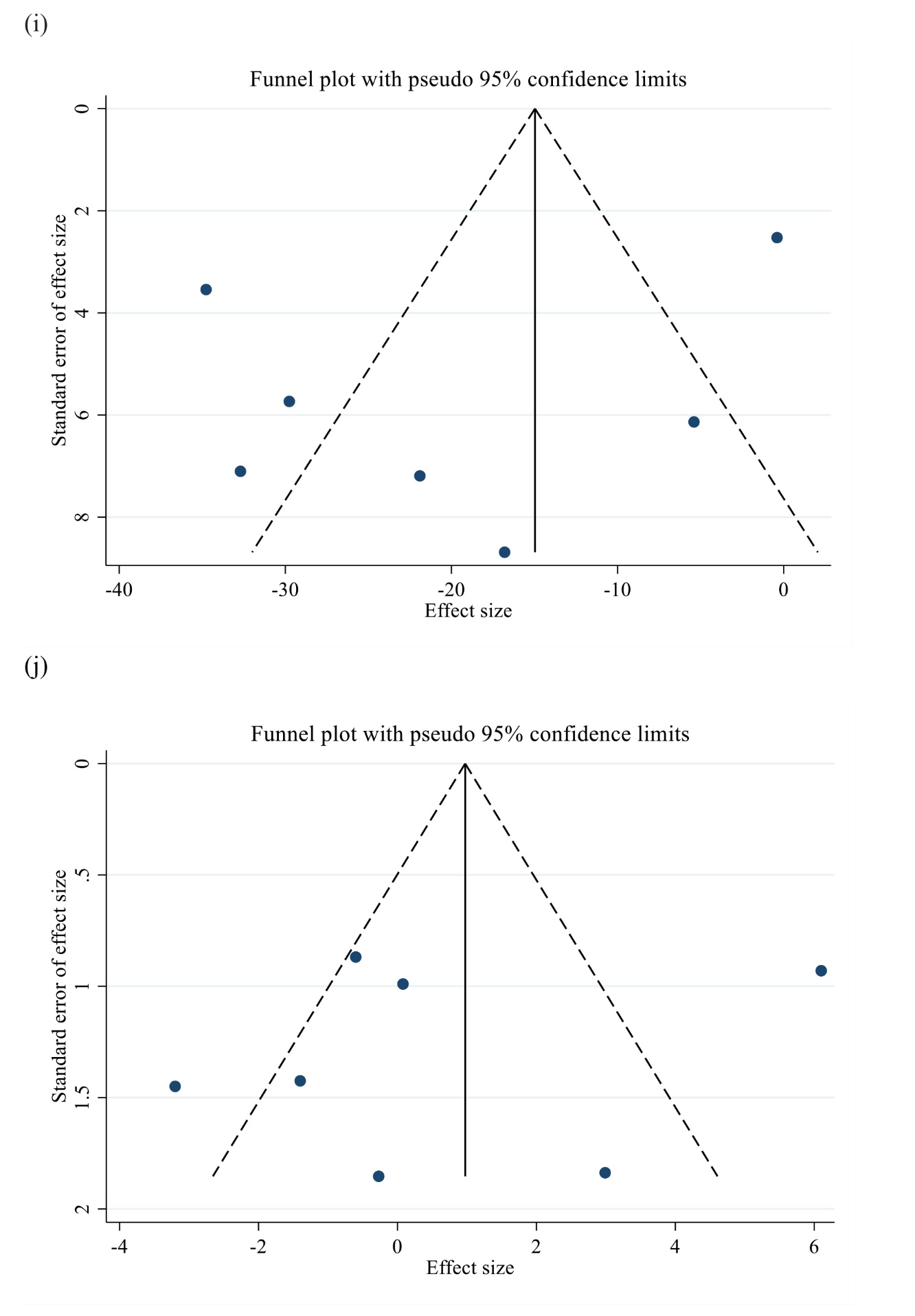


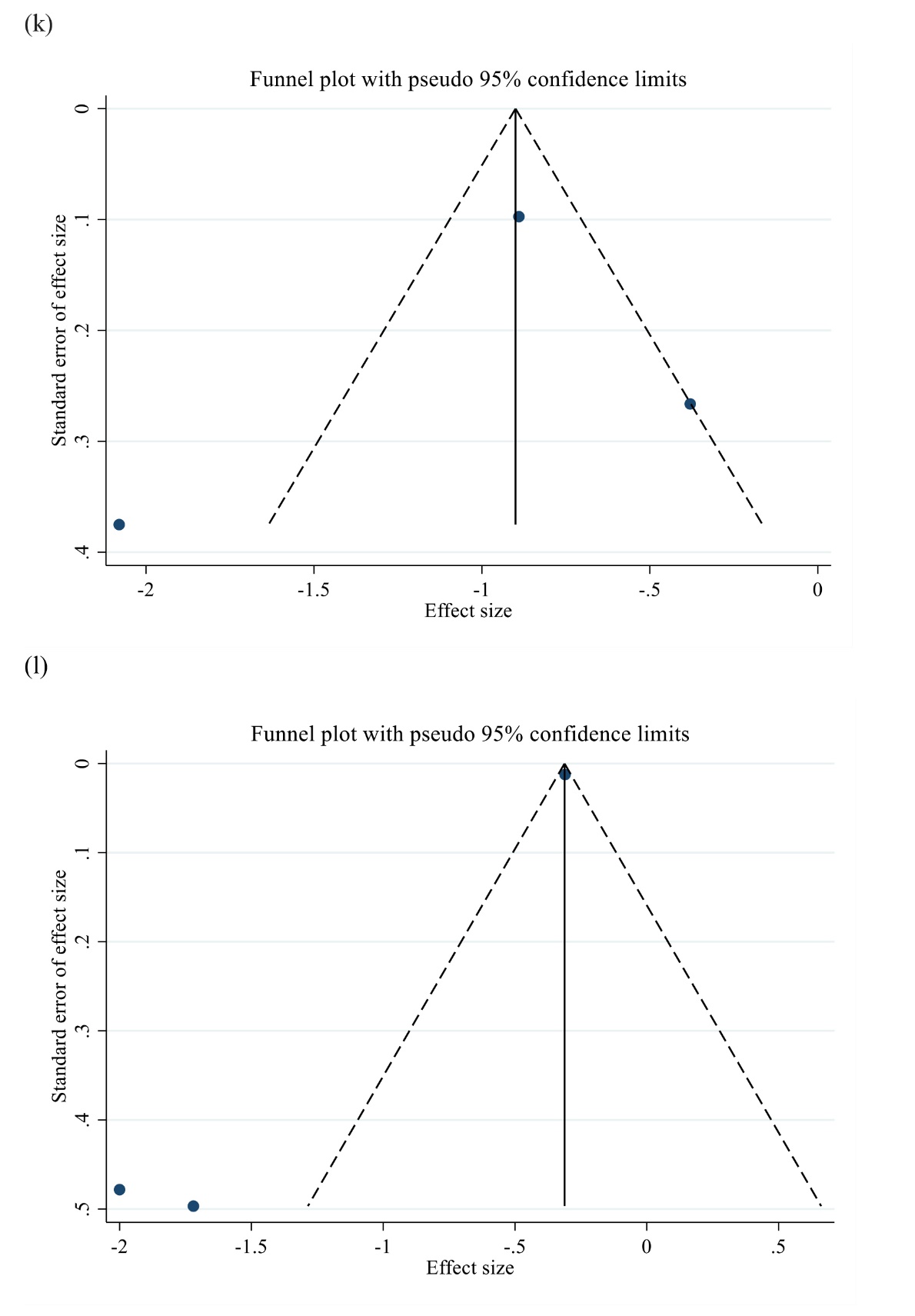


a: body mass index (BMI); b: fasting plasma glucose (FPG); c: hemoglobin A1c (HbA1c); d: oral glucose tolerance test (OGTT); e: fasting insulin; f: homeostatic model assessment of insulin resistance (HOMA-IR); g:; g: triglyceride (TG); h: total cholesterol (TC); i: low-density lipoprotein cholesterol (LDL-C); j: high-density lipoprotein cholesterol (HDL-C); k: C-reactive protein (CRP); l: Malondialdehyde (MDA).

**Supplementary Table 1.** Search terms used across the various databases

| PubMed | ("Black cumin"[Title/Abstract] OR "Nigella sativa"[Title/Abstract] OR "black seed"[Title/Abstract] OR "black caraway"[Title/Abstract] OR "Roman coriander"[Title/Abstract] OR "kalonji"[Title/Abstract] OR " fennel flower"[Title/Abstract] OR " pungent seeds"[Title/Abstract]) AND ("Type 2 diabetes"[Title/Abstract] OR T2DM[Title/Abstract] OR diabetes[Title/Abstract] OR "gestational diabetes mellitus"[Title/Abstract] OR GDM[Title/Abstract] OR prediabetes[Title/Abstract] OR prediabetic[Title/Abstract]) |
| --- | --- |
| Scopus | ( TITLE-ABS-KEY ( "Black cumin" OR "Nigella sativa" OR "black seed" OR "black caraway" OR "Roman coriander" OR "kalonji" OR " fennel flower" OR " pungent seeds" ) AND TITLE-ABS-KEY ( "Type 2 diabetes" OR t2dm OR diabetes OR "gestational diabetes mellitus" OR gdm OR prediabetes OR prediabetic ) ) |
| Web of science | "Black cumin" OR "Nigella sativa" OR "black seed" OR "black caraway" OR "Roman coriander" OR "kalonji" OR " fennel flower" OR "pungent seeds" (Topic) and "Type 2 diabetes" OR T2DM OR diabetes OR "gestational diabetes mellitus" OR GDM OR prediabetes OR prediabetic (Topic) |

**Supplementary Table 2.** Risk of bias assessment

| **studies** | **Random sequence generation** | **Allocation concealment** | **Selective reporting** | **Other sources of bias** | **Blinding (participants and personnel)** | **Blinding (outcome assessment)** | **Incomplete outcome data** | **General risk of bias** |
| --- | --- | --- | --- | --- | --- | --- | --- | --- |
| Najmi et al. 2012 | L | L | H | H | L | U | L | M |
| Hosseini et al. 2013 | L | L | L | H | L | U | L | L |
| Kaatabi et al. 2015 | L | L | H | H | H | U | L | H |
| Heshmati et al. 2015 | L | L | L | L | L | U | L | L |
| Ansari et al. 2017 | L | L | H | H | U | U | L | M |
| Hadi et al. 2018 | L | L | L | L | L | U | L | L |
| Kooshki et al. 2020 | L | L | H | H | L | U | L | M |
| Hadi et al. 2020 | L | L | L | L | L | U | L | L |
| Mostafa et al. 2021 | L | L | L | H | U | U | L | L |
| Jangjo-Borazjani et al. 2021 | L | L | L | H | L | U | L | L |
| Rahmani et al. 2022 | L | L | H | H | L | U | L | M |

*General Low Risk<2 high risk, General moderate risk=2 high risk, General high risk>2 high risk

**Supplementary Table 3.** Publication bias assessment

| BIAS | | |
| --- | --- | --- |
|  | Begg's Test | Egger's test |
| BMI | 1.000 | 0.282 |
| FPG | 1.000 | 0.831 |
| HbA1c | **0.016** | **0.005** |
| OGTT | 0.308 | **0.006** |
| Fasting insulin | 0.806 | 0.814 |
| HOMA-IR | 0.462 | 0.566 |
| TG | 0.133 | 0.467 |
| TC | 0.133 | **0.010** |
| LDL | 0.764 | 0.311 |
| HDL | 1.000 | 0.571 |
| CRP | 1.000 | 0.787 |
| MDA | 1.000 | 0.070 |

Abbreviations: BMI, body mass index; FPG, fasting plasma glucose; HbA1c, hemoglobin A1c; OGTT, oral glucose tolerance test; HOMA-IR, homeostatic model assessment of insulin resistance; TG, triglyceride; TC, total cholesterol; LDL-C, low-density lipoprotein cholesterol; HDL-C, high-density lipoprotein cholesterol; CRP, C-reactive protein; MDA, Malondialdehyde.
